# Supplementary material for: Bioprinting microporous functional living materials from protein-based core-shell microgels
Source: Nat Commun. 2023 Jan 19;14:322. doi: 10.1038/s41467-022-35140-5 (PMC9852579; doi:10.1038/s41467-022-35140-5)
Supplement: Supplementary file 3 — Description for Additional Supplementary Files [file 41467_2022_35140_MOESM3_ESM.docx]

**Description of supporting information**

Supplementary movie 1: Annealed microgel scaffold after incubation.

Supplementary movie 2: Extrusion printing microgels.
