# Supplementary material for: Bioprinting microporous functional living materials from protein-based core-shell microgels
Source: Nat Commun. 2023 Jan 19;14:322. doi: 10.1038/s41467-022-35140-5 (PMC9852579; doi:10.1038/s41467-022-35140-5)
Supplement: Supplementary file 6 — Reporting Summary [file 41467_2022_35140_MOESM6_ESM.pdf]

nature portfolio

Corresponding author(s): Ziyi Yu, Tuomas P.J. Knowles

Last updated by author(s): Nov 14, 2022

Reporting Summary

Nature Portfolio wishes to improve the reproducibility of the work that we publish. This form provides structure for consistency and transparency in reporting. For further information on Nature Portfolio policies, see our Editorial Policies and the Editorial Policy Checklist.

Statistics

For all statistical analyses, confirm that the following items are present in the figure legend, table legend, main text, or Methods section.

n/a	Confirmed	
☐	☒	The exact sample size (*n*) for each experimental group/condition, given as a discrete number and unit of measurement
☐	☒	A statement on whether measurements were taken from distinct samples or whether the same sample was measured repeatedly
☐	☒	The statistical test(s) used AND whether they are one- or two-sided<br>*Only common tests should be described solely by name; describe more complex techniques in the Methods section.*
☒	☐	A description of all covariates tested
☒	☐	A description of any assumptions or corrections, such as tests of normality and adjustment for multiple comparisons
☐	☒	A full description of the statistical parameters including central tendency (e.g. means) or other basic estimates (e.g. regression coefficient) AND variation (e.g. standard deviation) or associated estimates of uncertainty (e.g. confidence intervals)
☐	☒	For null hypothesis testing, the test statistic (e.g. *F*, *t*, *r*) with confidence intervals, effect sizes, degrees of freedom and *P* value noted<br>*Give P values as exact values whenever suitable.*
☒	☐	For Bayesian analysis, information on the choice of priors and Markov chain Monte Carlo settings
☒	☐	For hierarchical and complex designs, identification of the appropriate level for tests and full reporting of outcomes
☒	☐	Estimates of effect sizes (e.g. Cohen's *d*, Pearson's *r*), indicating how they were calculated

Our web collection on statistics for biologists contains articles on many of the points above.

Software and code

Policy information about availability of computer code

Data collection	No unique software or custom software was used to collect data and manufacturer softwares were used.<br>Images were acquired by OLYMPUS cellSens Eentry (v 2.3) and Leica LAX-S.<br>Rheology data was acquired by HAAKE RheoWin Measuring and Evaluation Software (v 4.80.0001) provided by the rheometer manufacturer.<br>Gas chromatography data was collected by ChemLab software (V.1.0.2.1), provided by the distributor of the GC equipment in China (Changzhou Panna Instrument).<br>High performance liquid chromatography data was collected by LabSolutions (v. 5.6) provided by the manufacturer (SHIMADZU).
Data analysis	Statistical analysis of numerical data was performed using Microsoft Excel 2019. Plotting was performed using Python (v. 3.8.12) scripts using NumPy (v. 1.22.1), Matplotlib (v. 3.5.1) libraries. Image processing was performed by FIJI-ImageJ (v 2.0.0-rc-69/1.52i). Adobe illustrator CC 2019 were used to design illustrations and curate data figures.

For manuscripts utilizing custom algorithms or software that are central to the research but not yet described in published literature, software must be made available to editors and reviewers. We strongly encourage code deposition in a community repository (e.g. GitHub). See the Nature Portfolio guidelines for submitting code & software for further information.

Data

Policy information about availability of data

All manuscripts must include a data availability statement. This statement should provide the following information, where applicable:

- Accession codes, unique identifiers, or web links for publicly available datasets
- A description of any restrictions on data availability
- For clinical datasets or third party data, please ensure that the statement adheres to our policy

All data generated in this study are provided in the Source Data file.

Human research participants

Policy information about studies involving human research participants and Sex and Gender in Research.

Reporting on sex and gender	N/A
Population characteristics	N/A
Recruitment	N/A
Ethics oversight	N/A

Note that full information on the approval of the study protocol must also be provided in the manuscript.

Field-specific reporting

Please select the one below that is the best fit for your research. If you are not sure, read the appropriate sections before making your selection.

☒ Life sciences ☐ Behavioural & social sciences ☐ Ecological, evolutionary & environmental sciences

For a reference copy of the document with all sections, see nature.com/documents/nr-reporting-summary-flat.pdf

Life sciences study design

All studies must disclose on these points even when the disclosure is negative.

Sample size	This work aims to examine the biocompatibility of the core-shell microgels for cell culture and to develop a new method for extrusion 3D bioprinting. For analysis of microbial and spheroids growth in microgels, no specific hypothesis was tested and no sample calculation, allocation of samples to different groups and randomization was performed. For these experiments, 30 uL cell-laden microgel suspensions were aliquoted and randomly placed under microscope for imaging. Spheroid growth data was from one representative experiment. For each sampling of spheroids, all spheroids imaged were analyzed (day 3 N=107, day 6 N=248). For microbial consortia experiments, the main hypothesis tested was through our method the bioactivities would increase. 3 independent scaffolds were printed for each experiments (bioremediation, scaffold degradation, and 2-PE fermentation) based on the laboratory experience and deemed to be sufficient to demonstrate such augmentation. Scaffold images were representative for macroscopic views. No statistical method was used to predetermine sample size but on the basis of previous publications: DOI: 10.1002/admt.201800632; DOI: 10.1039/c6lc00261g; DOI: 10.1039/c6lc00231e; doi.org/10.1038/s41467-020-14371-4; DOI: 10.1126/sciadv.aao6804.
Data exclusions	No data acquired for quantitative analysis were excluded.
Replication	The majority of experimentally conditions were independently repeated three times. All attempts at repetition were successful.
Randomization	Cells were randomly delivered to the droplets. Cell-laden microgels were randomly aliquoted for imaging and analysis. Cell-laden microgels were randomly assembled and annealed into scaffolds.
Blinding	For microbial consortia experiments, the experimenter for measuring the concentrations of chemicals using GC and HPLC were blinded as the samples were sent to other labs for analysis and labeled without any references to the samples. For other experiments, the main authors were not blinded. However, all experimental samples were collected and analyzed under the same condition, with the data analysis being conducted with the same software settings.

Reporting for specific materials, systems and methods

We require information from authors about some types of materials, experimental systems and methods used in many studies. Here, indicate whether each material, system or method listed is relevant to your study. If you are not sure if a list item applies to your research, read the appropriate section before selecting a response.

Materials & experimental systems

n/a	Involved in the study
☒	☐ Antibodies
☐	☒ Eukaryotic cell lines
☒	☐ Palaeontology and archaeology
☒	☐ Animals and other organisms
☒	☐ Clinical data
☒	☐ Dual use research of concern

Methods

n/a	Involved in the study
☒	☐ ChIP-seq
☒	☐ Flow cytometry
☒	☐ MRI-based neuroimaging

Eukaryotic cell lines

Policy information about cell lines and Sex and Gender in Research

Cell line source(s)	HEK 293T and A549 were obtained from ATCC.
Authentication	HEK 293T and A549 were authenticated at the time of purchase by short tandem repeats profiling.<br>No further authentication was performed by the authors of this work.
Mycoplasma contamination	All cell lines used were routinely tested for mycoplasma and the results were negative.
Commonly misidentified lines<br>(See ICLAC register)	Cell lines used were not present in the ICLAC register.
